# A Novel Idiopathic Atrial Calcification: Pathologic Manifestations and Potential Mechanism

**DOI:** 10.3389/fcvm.2022.788958

**Published:** 2022-03-21

**Authors:** Bowen Li, Qingbo Liu, Xihui Chen, Tangdong Chen, Wenhui Dang, Jing Zhao, Guangbin Cui, Kun Chen, Yuanming Wu

**Affiliations:** ^1^Department of Biochemistry and Molecular Biology, Air Force Medical University, Xi'an, China; ^2^Shaanxi Junda Forensic Medicine Expertise Station, Air Force Medical University, Xi'an, China; ^3^Department of Radiology & Functional and Molecular Imaging Key Lab of Shaanxi Province, Tangdu Hospital, Air Force Medical University, Xi'an, China; ^4^Department of Anatomy, Histology and Embryology and K.K. Leung Brain Research Centre, Air Force Medical University, Xi'an, China

**Keywords:** idiopathic atrial calcification, cardiac calcification, rare disease, autopsy, PPI, ABCC6

## Abstract

**Background:**

Cardiac calcification is a type of ectopic pathologic calcification of unknown etiology and mechanisms. Once diagnosed, the location, extent and morphology of the calcified lesions, as well as their functional significance in the heart, are usually the focus of case reports. Calcification is mostly distributed in myocardium, but rarely reported in atrium. In addition, because of limited sampling and complex pathological mechanisms, the etiology underlying the formation of these calcified lesions also remains unclear.

**Methods:**

Two cardiac calcifications were found in a patient, died of trauma-induced subarachnoid hemorrhage after slightly drinking, during a standard autopsy. The location and morphological characteristics of the calcified lesions were determined by computed tomography (CT) and CT-based 3D reconstruction. The specific histopathological characteristics of the lesions were determined by multi-staining. The concentration of free calcium and inorganic pyrophosphate (PPi) in plasma reflected the change of calcium metabolism. The expression and membranal localization of the ATP Binding Cassette Subfamily C Member 6 (ABCC6) in hepatocytes were detected by immunofluorescence. The variants of the ABCC6 were detected by Sanger sequencing and potential pathogenic variants were further identified by *in silico* analysis.

**Results:**

The present study describes a patient with idiopathic calcification with two pear-shaped and irregularly hollow lesions symmetrically distributed in the patient's atrium. Massive accumulation of calcium salts was identified by multi-staining. For this patient, the plasma concentration of free calcium was higher than the control, indicating that calcium metabolism was disturbed. Furthermore, the plasma PPi of the patient was lower than the normal. By using immunofluorescence, the expression and membranal localization of ABCC6 was decreased and impaired in hepatocytes, respectively. Combined with Sanger sequencing and *in silico* analysis, 7 variants were identified.

**Conclusions:**

This study described a novel patient with symmetrically distributed idiopathic atrial calcifications. Furthermore, all the results indicated that these pathologic calcifications may be secondary to reduced plasma PPi content due to ABCC6 dysfunction in hepatocytes. Moreover, these findings provided novel clues to the pathogenesis, clinical diagnosis and treatment of idiopathic atrial calcification in future.

## Introduction

Cardiac calcification, a rare type of cardiac pathology, is characterized by the abnormal accumulation of calcium salts in the heart (1). Two basic forms of cardiac calcification have been described, dystrophic and metastatic. Dystrophic calcification is a consequence of injury to cardiac tissue, such as necrosis or degeneration due to heart failure, arrhythmia, ischemic heart disease or cardioembolic disease, whereas metastatic calcification results from an imbalance in calcium-phosphate homeostasis triggered by primary hyperparathyroidism, renal failure, vitamin D deficiency, hypervitaminosis D or inflammatory processes (2–5). Although idiopathic calcification may be a third type of cardiac calcification, the clinical validity and utility of this classification remain unclear.

The etiology, prevalence and specific mechanisms of idiopathic calcification of the heart are unknown (6). Studies to date have been limited to case reports describing the medical history of patients with idiopathic cardiac calcification and the histopathology of this condition. Idiopathic calcification may be a type of dystrophic or metastatic calcification secondary to clinically remote or occult pathological processes (2). Patients with idiopathic cardiac calcifications often present with massive accumulation of calcium salts in the ventricles, with or without involvement of the great vessels, such as the coronary arteries, and pericardium. Because of the limited numbers of patients assessed to date and the complex pathological mechanisms of this condition, most reports to date on patients with idiopathic cardiac calcification have described their cardiac function, as well as the physiological location, extent and pathological characteristics of calcified foci (2). By contrast, few studies have evaluated the molecular mechanism underlying this condition.

Circulating inorganic phosphate (PPi) has been identified as a key endogenous inhibitor of biomineralization and ectopic calcification (7–9). Plasma PPi is mainly generated by the hydrolyzation of extracellular ATP released from hepatocytes. The pumping of ATP from intracellular to extracellular are mediated primarily by ABCC6 of hepatocytes (7). Variants or other types of defects in ABCC6 lead to ectopic calcification (10–12). In addition, variants in ABCC6 may affect its membrane localization, leading to functional impairment (13). However, the relation between hepatocyte ABCC6 plasma PPi and cardiac calcification is still unclear.

The present study describes a novel patient with symmetrically distributed idiopathic calcifications of the left and right atria. The location and morphological characteristics of the calcified lesions were determined by computed tomography (CT) and CT-based 3D reconstruction, and the specific histopathological characteristics of these lesions were described by multi-staining. To explore possible molecular mechanisms, the concentrations of calcium and PPi were measured in the patient's plasma. A lower plasma concentration of PPi may have been due to a dysfunction in hepatocyte ABCC6. These findings not only suggested a molecular mechanism responsible for idiopathic calcification, but also provided clues to methods that could prevent cardiac calcification.

## Materials and Methods

### Autopsy and Heart Imaging

Autopsy examination was organized by Shaanxi Junda Forensic Medicine Expertise Station according to the national autopsy standards strictly and was carried out with Research Ethics Board approval from the XiJing Hospital of the Fourth Military Medical University. Written informed consents have been obtained from the next of kin for the autopsy examination. The forensic certificate was derived from autopsy examination and histological examination of pathological specimens. For heart imaging, 64-detector scanner computed tomography (CT, Sensation 64, Siemens, Erlangen, Germany) was used to yielding an isotropic 3D data set. 3D reconstruction of the set was created by using RadiAnt DICOM Viewer (64-bit). The calcifications signal and angle were obtained by manual adjustment.

### Histopathology of Calcified Lesion

Formalin-fixed, methyl methacrylate (MMA)- embedded cardiac lesions (100 μm thick) were cut using a hard tissue sectioning system (Leica, Cat #SP1600, German). Hematoxylin and eosin staining was used for the light-microscopic studies. Masson's trichrome staining was applied to identify the myocardial fibrosis. The Alizarin red S staining was devoted to detect the calcification in myocardium. Safranin O-Fast Green staining was employed to distinguish the osteogenesis and cartilage. All these histopathological studies were supplied by Servicebio (Wuhan, China). All the images were taken by the Olympus VS200 (Japan).

### Plasma Free Calcium Detection

Blood samples, drawn within 12 h of death, were collected in K2 EDTA BD Vacutainer tubes and processed within 1 h of collection. The whole blood sample were centrifuged at 820 g for 10 min at room temperature. The supernatant was further centrifuged at 16,000 g for 10 min to pellet remaining cellular debris and then stored at −80°C. The plasma free calcium levels were detected by using an enzyme labeling assay-based plasma free calcium concentration detection kit (Solarbio, Cat #BC0720, Beijing, China). The samples from control group were collected from 7 healthy male with the same age, who were all died of trauma. For these samples, the absorbance at 520 nm were measured by microplate reader (TECAN, Cat #spark).

### Detection of Pyrophosphate in the Plasma

For the pyrophosphate detection, the whole blood sample were centrifuged at 820 g for 10 min at room temperature. The supernatant was further filtrated through a Centrisart I mass cutoff filter (Sartorius) (2,200 g for 20 min at 4°C) to deplete platelets. Then, the plasma sample were stored at −80°C (7). The plasma pyrophosphate levels were measured by a fluorescent assay using a PhosphoWorks™ Fluorimetric Pyrophosphate Assay Kit (AAT Bioquest, Cat # 21611) following the manufacturer's instructions. The samples from control group were collected from 7 healthy male with the same age, who were all died of trauma. The fluorescence (excitation:316 nm, emission: 456 nm) of the samples were measured by microplate reader (TECAN, Cat #spark).

### Immunofluorescence Staining

Tissues were fixed in formalin and paraffin-embedded. Liver from patient and control were cut into 4 μm-thick sections. The samples from control group were collected from 7 healthy male with the same age, who were all died of trauma. For one sample, two sections were stained with anti-ABCC6 (Proteintech, Cat #27848-1-AP, 1:50) for overnight in 4°C. After washing twice by PBS, the sections were stained with second antibody FITC-labeled goat anti-rabbit (Servicebio, Cat #GB23303, 1:500). All the images were taken by Olympus VS200. The average fluorescence intensity of ABCC6 was analyzed by Fiji Image J (NIH, Bethesda, MD, United States).

### Sanger Sequencing and *in silico* Analysis of *ABCC6*

The DNA of this patient was extracted from blood sample by using QIAamp®DNA mini Kit (QIAGEN, Cat #51304, Germany). The 31 exons of ABCC6 were amplified by high-fidelity polymerase chain reaction (PCR). The specific primer sequences are summarized in Supplementary Table S1. Sanger sequencing is completed by Tsingke Biotechnology. The sequencing results are exhibited by SnapGene®4.1.9. Further *in silico* analysis is achieved by Franklin tools (https://franklin.genoox.com/).

### Statistics Analysis

All data were presented as mean ± SD. Graphical representation of the data were performed using GraphPad Prism 8.3 (GraphPad Software, San Diego, CA).

## Results

### Case Report

A 40-year-old man with no previous medical history of central nervous system disorders became comatose and tumbled after drinking alcohol and died after half an hour of unconsciousness. An autopsy examination was performed to determine the cause of death (14).

The autopsy examination and pathological section revealed that a trauma-induced subarachnoid hemorrhage after slightly drinking (ethanol concentration in blood: 5.88 mg/100 mL) was the direct cause of death (Supplementary Figure S1A). There was no obvious hemorrhage or necrosis in the brain stem. Examination of the body showed no evidence of cerebrovascular diseases, especially aneurysms, malformations or other lesions in vertebral arteries. No pathological features of fatal diseases were found in the liver, kidneys, spleen, pancreas or other visceral organs. Examination of the cardiovascular system showed no evidence of significant coronary stenosis, the pathological characteristics of acute or chronic ischemia, or necrosis of the myocardium (Supplementary Figures S1B–H). By investigating the patient's lifestyle, the patient did not have the history of alcohol addiction.

**Figure 1:**
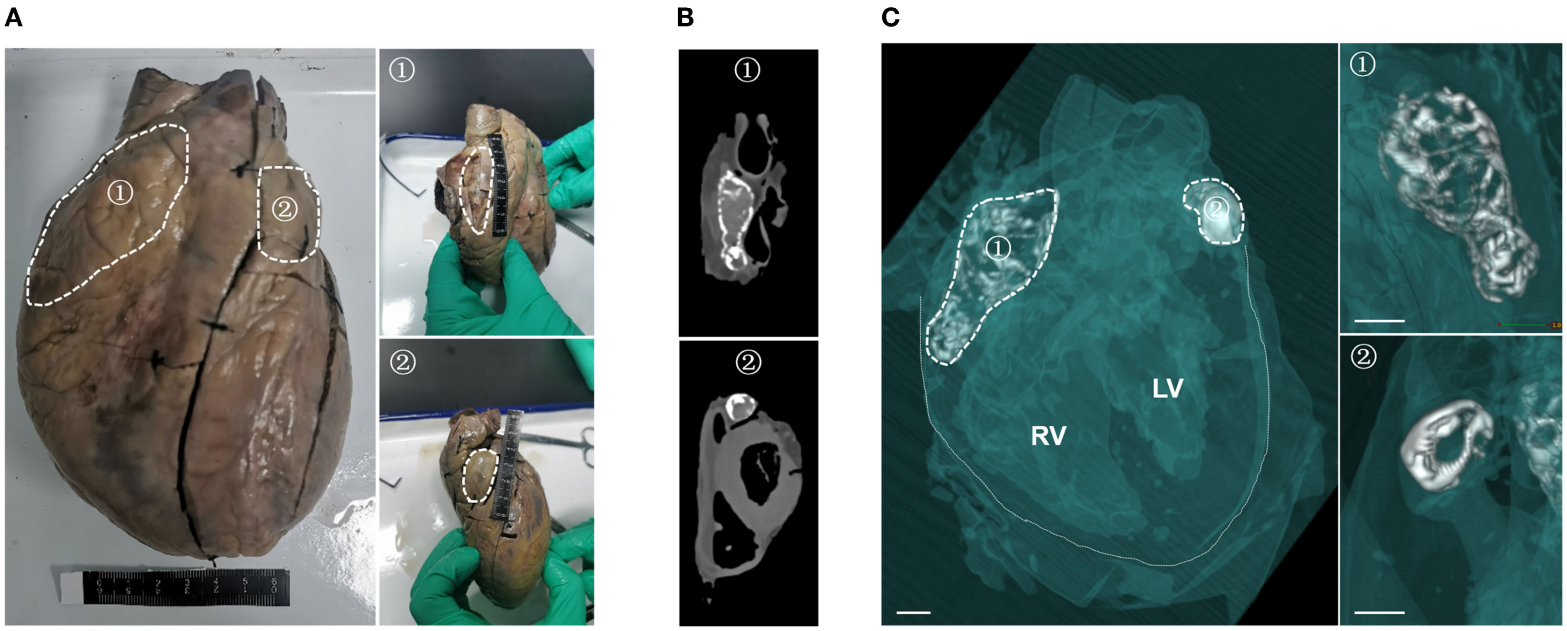
Symmetrical distribution of two calcifications in the left and right atria of this patient. **(A)** Gross examination of the heart, showing two hard, palpable calcifications in the left (②) and right (①) atrial chambers. White dashed lines show the outlines of the two calcifications. **(B)** Computed tomography scan showing the two calcifications in the patient's heart. **(C)** 3D reconstruction of the heart specimen from the patient from the left oblique frontal view. Soft tissue (myocardium, congestion and blood vessel) is semitransparent green in color, calcium density is white and air density is clear. The solid white lines in the left panel delineate the boundaries of the heart, and the white dashed lines delineate the outlines of the two calcifications. Structural details of the two calcifications are magnified in the right panel. Scale bar = 1 cm.

Surprisingly, during the autopsy, two hard objects, one large (5 cm long) and one small (2.5 cm long), were detected at the outlets of the left and right coronary arteries, respectively (Figure 1A). The heart was found to weigh 359 g; each atrium and ventricle was intact, and none of the valves showed thickening, stenosis or other abnormalities. No dilation was observed in the bilateral ventricular chambers. There was no evidence of thickening of the flesh column, the epicardium and intima were smooth, and no abnormalities were observed. Stenosis was not present in the coronary lumen, and atherosclerosis was not observed in the wall. CT-based 3D reconstruction showed that the two hard objects were of irregularly hollow shape and were symmetrically distributed in the patient's left and right atria (15, 16) (Figures 1B,C). In addition, there were no calcifications in myocardial tissue (especially the ventricular muscle) or in the arteries and veins, and no significant narrowing of the coronary arteries.

These findings indicated that this patient might present a particular form of cardiac calcification. To assess the etiology and pathological features of the different classifications of cardiac calcification and to determine the calcification type of this patient, case reports of patients with cardiac calcification published in the past 20 years were reviewed (Table 1). By comparing the gender, age, etiology, and location of calcifications in the literature, we concluded that the patient might have idiopathic atrial calcification, a novel form of cardiac calcification symmetrically distributed in both atria.

**Table 1:** Demographic and clinical characteristics of patients diagnosed with cardiac calcification.

**Classification**	**Gender**	**Age**	**Etiology**	**Distribution of the calcification**	**Published date**	**References**
Dystrophic calcification	Male	71 years	Stenosis of the coronary artery	Papillary muscle	2001	(17)
	Female	20 years	Septic shock	Midmyocardium of the entire left ventricle	2002	(18)
	Unknown	40 years	Thoracic radiation and cardiomyopathy	Extensive left atrium	2004	(19)
	Female	46 years	Stenosis of the coronary artery	Left ventricular myocardium, interventricular and interatrial septae	2006	(20)
	Male	65 years	Old myocardial infarction	Apex of the heart	2006	(21)
	Male	70 years	Myocardial infarction	Curvilinear focal apical left ventricular calcification	2007	(22)
	Male	5 days	Acute neonatal myocarditis due to Coxsackie virus type B	Diffuse myocardial calcification	2009	(23)
	Male	67 years	Coronary artery calcification	Spindle-like extensive calcification in anterolateral papillary muscle	2011	(24)
	Male	56 years	Perimyocarditis in the setting of Shigella sepsis	Diffuse calcification	2011	(25)
	Male	2 weeks	Fetal infective endocarditis	Postnatal tricuspid valve and pulmonary valve	2013	(26)
	Male	69 years	Trauma	Left ventricular wall	2015	(27)
	Male	65 years	Weber Christian disease	Lateral wall of the left ventricular myocardium	2015	(28)
	Male	32 years	Acute myocarditis	Extensive myocardial calcification in the middistal septum	2015	(29)
	Male	60 years	Myocardial infarction	Apex of the left ventricle	2016	(3)
	Female	89 years	Mitral annulus calcification	Severe continuous linear caseous calcification from the mitral annulus expanding to the contiguous ventricular myocardium till the apex	2017	(30)
	Male	18 years	Klinefelter syndrome	Dense calcification of the left and right ventricular myocardium	2018	(31)
	Male	17 years	Epstein–Barr viral myocarditis	Left ventricular wall	2018	(32)
	Female	21 years	Septic shock	Diffuse punctate myocardial calcifications, involving interventricular septum, left ventricle and the anterior margin of the right atrium and ventricle	2019	(33)
	Male	15 years	Fulminant myocarditis	Diffuse in both ventricles	2019	(34)
	Female	41 years	Septic shock	Left ventricular wall	2019	(35)
	Female	33 years	Septic shock	Left ventricular wall	2020	(36)
	Male	45 years	Septic shock	Left ventricular wall	2020	(37)
	Female	36 years	Septic shock	Contraction band necrosis with minimal endocardial amyloid and patchy myocardial calcification	2020	(38)
	Female	43 years	Septic shock	Left ventricular wall	2020	(39)
	Female	67 years	Traumatic damage	Interventricular septum	2021	(5)
	Male	51 years	Septic shock	Diffuse left ventricular mid-myocardial calcification	2021	(40)
Metastatic calcification	Female	34 years	End-stage renal disease	Coronary artery and left ventricular myocardium	2012	(41)
	Male	47 years	Hemodialysis-dependent end-stage renal disease	Anterior massive myocardial calcification	2012	(42)
	Male	53 years	End-stage renal disease	Extensive myocardial calcification involving the left ventricle and interventricular septum	2018	(14)
	Female	54 years	Hyperparathyroidism	Basal interventricular septum, anterior walls and posterolateral walls of the left ventricle	2018	(43)
	Male	24 years	Thrombocytopenia, anasarca, fever, renal insufficiency or reticulin fibrosis, organomegaly (TAFRO) syndrome	Bilateral ventricular walls	2021	(44)
	Female	infant	Subcutaneous fat necrosis of the newborn (SCFN)	Atrial myocardial calcification	2003	(45)
	Female	42 years	Systemic hypercalcemia	Diffuse myocardial calcification	2005	(46)
	Female	6 years	Prolonged history of dietary deficiency of calcium and vitamin D	Extensive cardiac calcifications	2005	(47)
Idiopathic calcification	Female	6.5 weeks	*ENPP1* variant	Multiple areas of calcification	2006	(48)
	Female	4.5 months	Unknown	Left ventricle	2006	
	Male	57 years	Unknown	Myocardium of the left ventricle, mitral annulus and left atrium wall and pulmonary veins	2012	(49)
	Female	56 years	Unknown	Left ventricle, widespread in a spiral pattern	2014	(50)
	Female	56 years	Unknown	Extensive myocardial calcifications in the left ventricle	2016	(51)
	Female	71 years	Unknown	Diffuse calcific infiltration of the left ventricular myocardium, involving the papillary muscles, mitral chordal apparatus and mitral annulus	2020	(52)
	Female	74 years	Unknown	Extensive infiltrative calcification of the atrioventricular groove, and uncommon intramyocardial calcification of the ventricular septum and inferior wall	2021	(53)
	Male	28 years	Unknown	Extensive myocardial calcifications in the left ventricle	2021	(54)
Unknown	Male	54 years	Atrial septal defect closure for 30 years	Over the posterior and diaphragmatic sides of the heart	2002	(55)
	Female	62 years	Unknown	Apex of the left ventricle	2009	(56)
	Female	81 years	Unknown	Interventricular septum, left ventricular wall and in the mitral annulus	2010	(57)
	Male	83 years	Unknown	Sharp calcified pericardial plaque	2012	(58)

*ENPP1, ectonucleotide pyrophosphatase-phosphodiesterase 1 gene*.

### Characterization of Histopathological Features in the Calcifications

**Figure 2:**
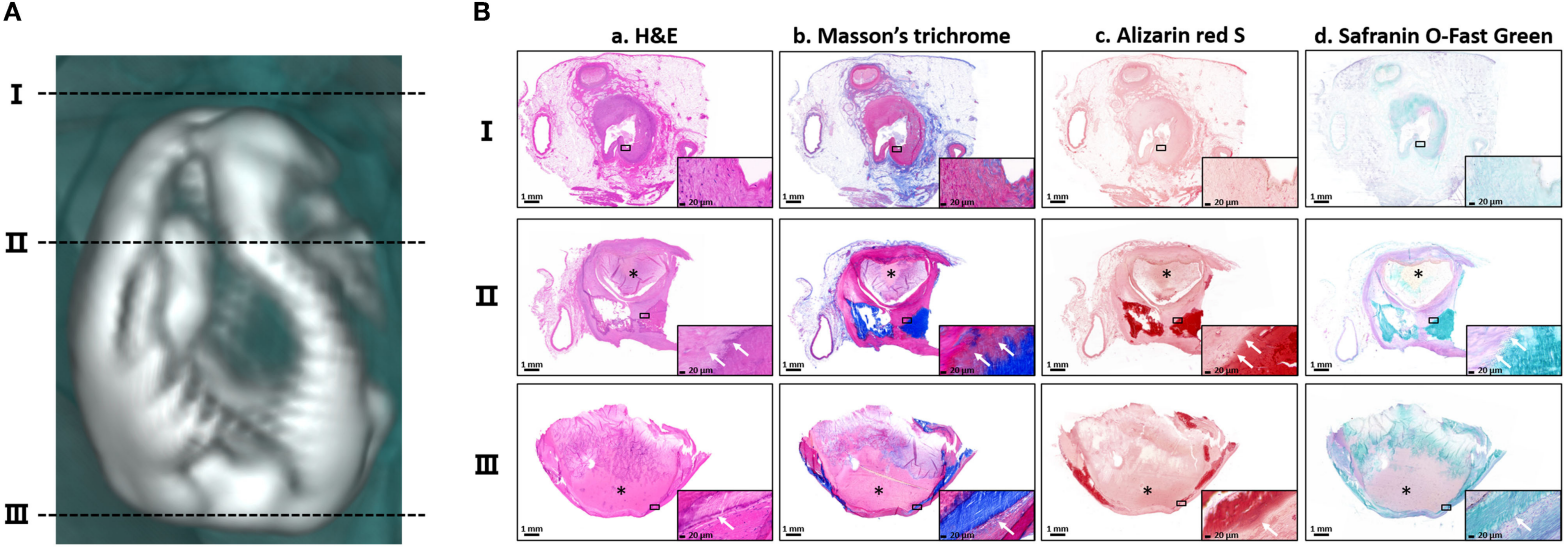
Histopathological features of the calcifications. **(A)** The three black dotted-lines indicate the positions of the selected horizontal sections of the calcification located in the left atrium. The corresponding numbers are shown on the left. **(B)** Staining of the three horizontal sections with (a) hematoxylin–eosin (H&E), (b) Masson's trichrome, (c) Alizarin red and (d) Safranin O-fast green. Magnified images are shown in the lower corner of each image. The asterisks indicate the thrombus. The white arrows indicate the pathological infiltration of calcium salts. Scale bars are shown at the bottom of each image.

To characterize the pathological features of the calcifications, the calcified lesion in the left atrium was chosen for histopathological staining. Because this lesion was pear-shaped and irregularly hollow, three horizontal sections (I, II and III) perpendicular to its long axis were selected for further histopathological analysis (Figure 2A) by multi-staining (59) (Figure 2B). Hematoxylin–eosin staining showed blue-purple aggregates suggestive of calcium signals interspersed within the lesion (Figures 2B-a). Staining of collagen fibers with Masson's trichrome showed that the calcified lesions may have replaced necrotic myofibers. However, some of the tissues near the lesion were heavily fibrotic, suggesting extensive remodeling of the atrial muscle (Figures 2B-b). Staining with the calcium-specific dye Alizarin red S confirmed the calcified nature of the lesions (Figures 2B-c). Because the lesion was hard, pathologically similar to bone and had a well-defined calcification boundary, the presence of ossifying cartilage tissue near the area of calcification was assessed. The calcified lesions were positively stained with Safranin O-fast green, a marker of osteogenesis. The adjacent area, however, was not stained red, a marker of cartilage, indicating that there was no cartilage around the lesion (Figures 2B-d). The pathological staining also demonstrated the infiltration of partial calcified lesions into soft tissue. In addition, the thrombus, marked by asterisks, was not directly adjacent to the calcifications (Figure 2B, Supplementary Figure S2B). Taken together, all these results elucidated the pathologic features of calcified lesions and demonstrated that the calcified lesions actually did not isolate to the left and right atrial appendages.

### Disturbed Metabolism of Ectopic Calcification Regulators in the Patient's Plasma

**Figure 3:**
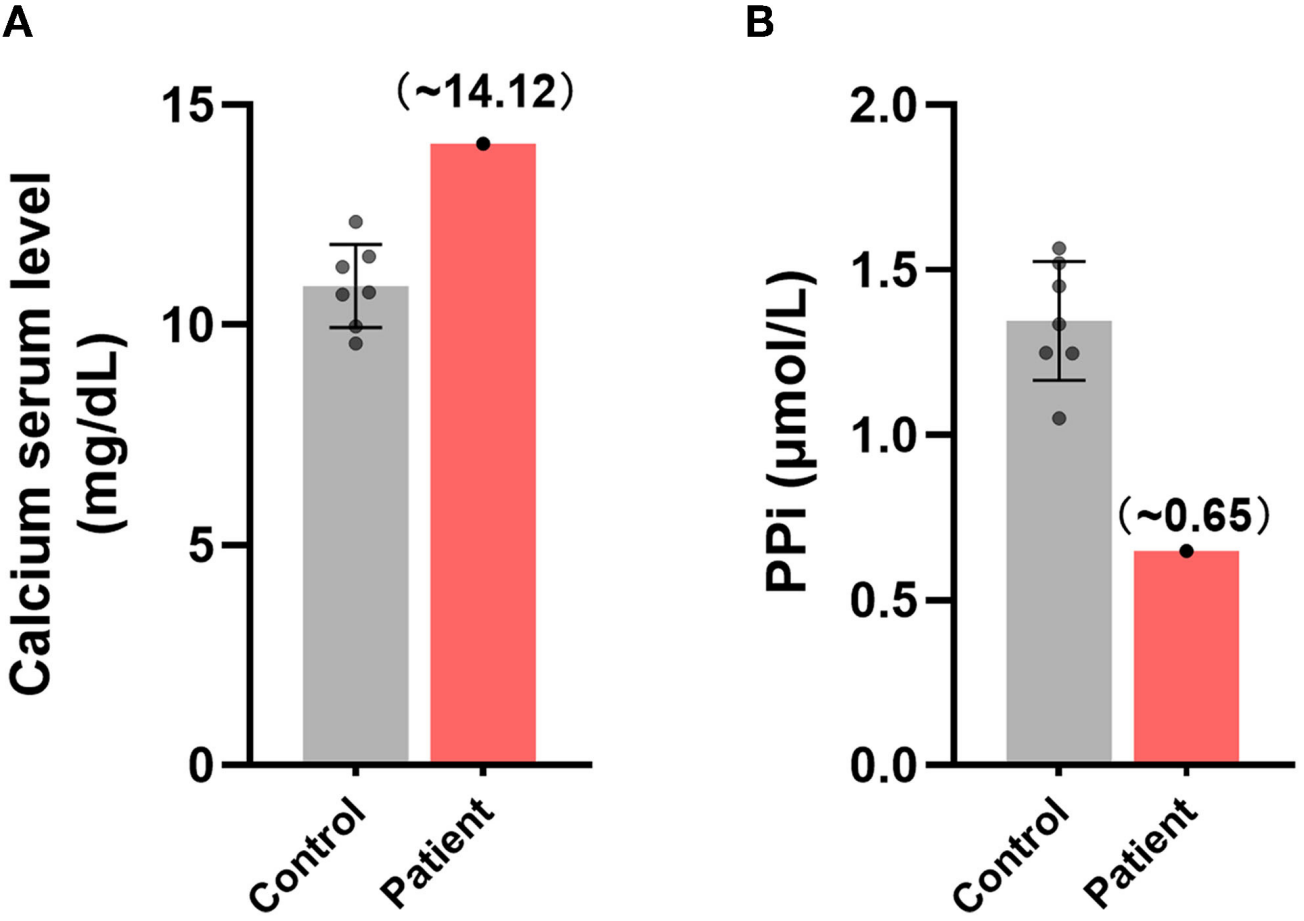
Disturbances in the metabolism of ectopic calcification regulators in the plasma of this patient. Comparisons of the relative plasma concentrations of **(A)** free calcium and **(B)** inorganic pyrophosphate in this patient and control group.

Idiopathic calcification is a type of ectopic calcification (2, 60), and disorders of calcium metabolism in the circulation significantly correlate with the appearance of an ectopic calcification phenotype (61). The plasma concentration of free calcium in this patient was found to be 14.12 mg/dL, higher than the normal range of 10.89 ± 0.88 mg/dL, suggesting that calcium metabolism in this patient's plasma was unbalanced (Figure 3A).

In consideration of the key role of circulating PPi in inhibition of endogenous biomineralization and ectopic calcification (7–9), we further measured the content of plasma PPi of the patient. The plasma PPi in this patient was 0.649 μmol/L, significantly lower than the normal concentration of 1.346±0.167 μmol/L in healthy individuals (Figure 3B). These findings suggested that imbalances in free calcium and PPi may have been associated with the development of idiopathic calcification in this patient.

### Idiopathic Calcification May Have Been Caused by ABCC6 Dysfunction in This Patient's Hepatocytes

**Figure 4:**
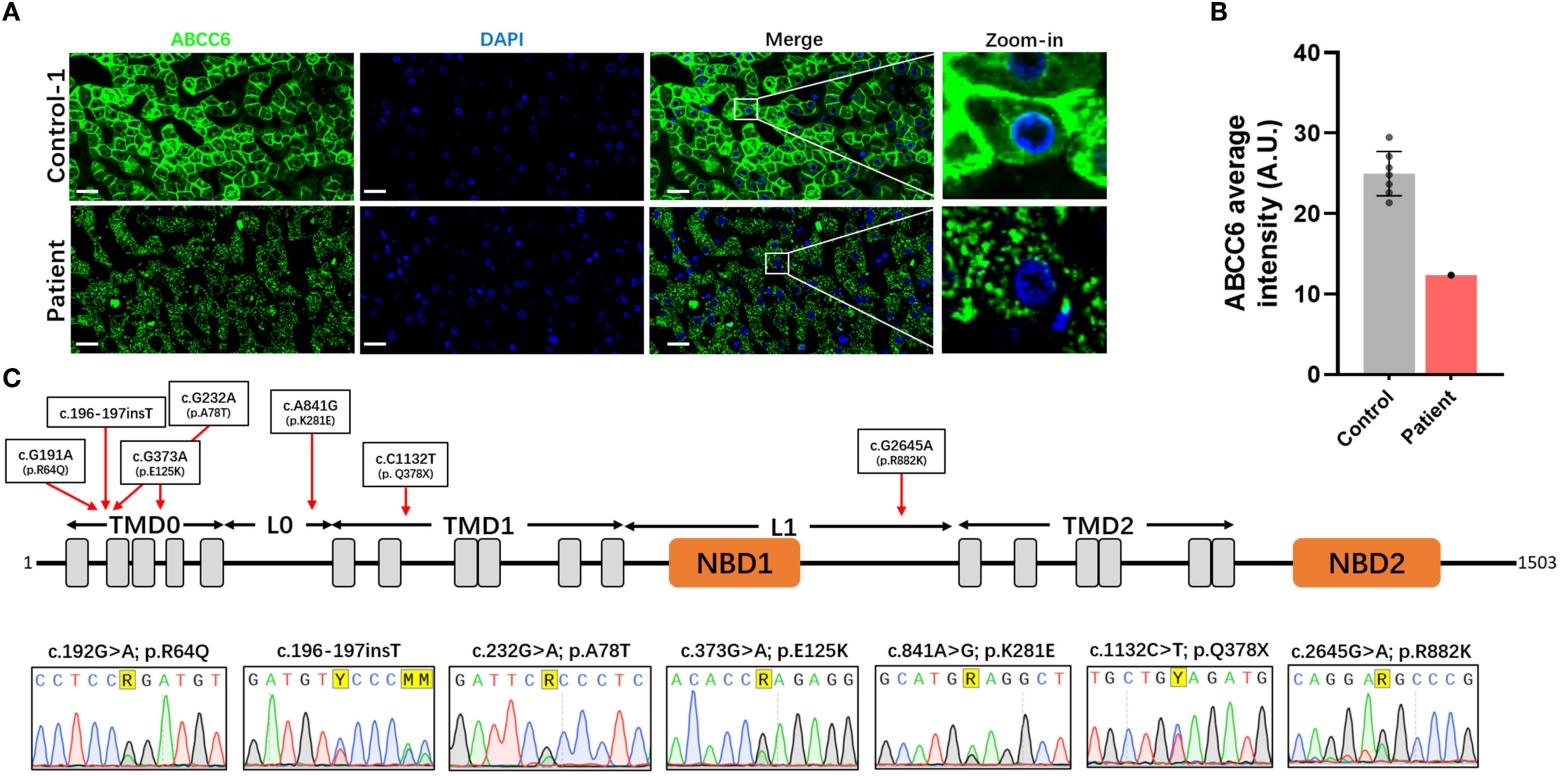
Dysfunction of ABCC6 was observed in the hepatocytes of this patient. **(A)** The immunofluorescence of the liver in control-1 and patient. Green indicated ABCC6. Blue indicated cell nucleus. Scale bar = 20 μm. **(B)** Average immunofluorescence intensity of hepatocellular ABCC6 in control group (A &) and patient. **(C)** The domain structure of ABCC6 and positions of identified patient variants were indicated in the up pattern. The Sanger sequencing results were shown under the structure. TMD, transmembrane domain; L, linker region; NBD, nucleotide binding domain.

To investigate whether the decrease of plasma PPi was caused by ABCC6 deficiency in hepatocytes, we further examined the distribution and content of ABCC6 in hepatocytes. Measurement of the relative level of expression and subcellular localization of ABCC6 in this patient using immunofluorescence (Figure 4A, Supplementary Figure S3) showed that the ABCC6 concentration in this patient was lower than in the control group (Figure 4B, Supplementary Figure S3), and that its membrane localization was significantly affected (Figure 4A).

Moreover, in using Sanger sequencing, we identified 7 heterozygous variants of ABCC6 (Figure 4C). These variants included 5 missense variants (c.191G>A [p.Arg64Gln], c.232G>A [p.Ala78Thr], c.373G>A [p.Glu125Lys], c.841A>G [p.Lys281Glu], c.2645G>A [p.Arg882Lys]), 1 insertion variant (c.196-197insT) and 1 nonsense variant (c.1132C>T [p.Gln378^*^]) (Figure 4C). The predicted topology of ABCC6 included three transmembrane domains (TMD0, TMD1, and TMD2), 2 linker regions (L0 and L1) and 2 nucleotide binding domains (NBD1 and NBD2) (Figure 4C). The distributions of these variants were indicated in Figure 4C.

Furthermore, according to further *in silico* analysis, we noticed that the insertion variant and the nonsense variant may contribute to the lower ABCC6 concentration of the patient's hepatocytes (Table 2). Moreover, since the L0 domain is crucial for the membrane localization of ABCC6 (62), the L0 domain-located missense variant (c.841A>G [p.Lys281Glu]) may trigger the membrane localization anomality of ABCC6 by affecting L0 folding. These findings indicate that the low plasma PPi levels in this patient were caused by ABCC6 variant or loss of function.

**Table 2:** *In silico* analysis of the patient derived *ABCC6* variants.

**Variant position** **(chromosome/position)**			**c.191G>A (16/16315534)**	**c.196-197insT** **(16/16315528)**	**c.232G>A (16/16313792)**	**c.373G>A** **(16/16313512)**	**c.841A>G (16/16297424)**	**c.1132C>T** **(16/16295902)**	**c.2646G>C (16/16269788)**
			**p.R64Q**	**-**	**p.A78T**	**p.E125K**	**p.K281E**	**p.Q378X**	**p.R882K**
	Aggregated	Aggregated prediction	Benign (0.06)	N/A	Benign (0.03)	Uncertain (0.44)	Uncertain (0.33)	N/A	Uncertain (0.29)
		Revel	Benign (0.06)	N/A	Benign (0.03)	Benign (low) (0.44)	Benign (low) (0.33)	N/A	Benign (low) (0.29)
		Eve	N/A	N/A	N/A	N/A	N/A	N/A	N/A
		Varity	Benign (0.06)	N/A	Benign (0.03)	Benign (low) (0.3)	Benign (0.05)	N/A	Benign (low) (0.12)
		MUT Assesor	Neutral (0.7)	N/A	Low (0.88)	Medium (3.15)	Neutral (-1.17)	N/A	Neutral (0)
		SIFT	Tolerated (0.13)	N/A	Tolerated (0.12)	Damaging (0.01)	Tolerated (1)	N/A	N/A
Predictions	Functional coding	Polyphen2	Benign (0.15)	N/A	Benign (0.02)	N/A	N/A	N/A	N/A
		MT	Deleterious (low) (0.51)	N/A	Benign (0)	Deleterious (1)	Benign (0)	Deleterious (1)	Benign (0.29)
		FATHMM	Benign (0.44)	N/A	Benign (1.31)	Benign (0.91)	Deleterious (-2.64)	N/A	N/A
		MetaLR	Benign (0.1)	N/A	Benign (0.05)	Benign (low) (0.27)	Benign (low) (0.26)	N/A	Benign (low) (0.46)
		dbscSNV Ada	N/A	N/A	N/A	N/A	N/A	N/A	N/A
		RF	N/A	N/A	N/A	N/A	N/A	N/A	N/A
	Functional whole genome	GenoCanyon	Benign (0.01)	N/A	Benign (0)	Benign (0.01)	Benign (0)	Deleterious (0.98)	Benign (0)
		fitCons	Deleterious (0.52)	N/A	Deleterious (0.55)	Benign (0.49)	Benign (0.5)	Deleterious (0.58)	Deleterious (0.55)
		gnomAD (Aggregated)	N/A	N/A	N/A	0.0776%	0.0021%	0.006%	N/A
		TOPMed Bravo	N/A	N/A	N/A	N/A	N/A	0.0172%	N/A
		GME Variome	N/A	N/A	N/A	N/A	N/A	N/A	N/A
		Iranome	N/A	N/A	N/A	N/A	0.0625%	N/A	N/A
Population frequencies		ExAC	0.0115%	N/A	N/A	N/A	0.0041%	0.0008%	N/A
		1000 Genomes	N/A	N/A	0.0799%	N/A	N/A	N/A	N/A
		ESP 6500	N/A	N/A	N/A	N/A	N/A	N/A	N/A
		4.7KJPN	N/A	N/A	N/A	N/A	N/A	N/A	N/A
		GenomeAsia	N/A	N/A	N/A	N/A	N/A	N/A	N/A
		Mexican DB	N/A	N/A	N/A	N/A	N/A	N/A	N/A
Suggested classification			VUS	Pathogenic	VUS	VUS	VUS	Pathogenic	VUS

*The symbol of “N/A” indicates that there have no data. The row of “Suggested classification” represents the description of pathogenicity of these variants according to the American College of Medical Genetics and Genomics (ACMG) guidelines. “VUS” indicates “uncertain significance.”*

## Discussion

Idiopathic cardiac calcification usually refers to a class of ectopic calcifications in heart of undetermined prevalence, etiology and mechanisms. The present study describes a novel case of idiopathic atrial calcification, characterized by calcified lesions symmetrically distributed in the patient's atria. CT-based 3D reconstruction showed that these lesions were irregular, and histopathological staining showed the deposition of calcium salts in these calcified lesions. The fibrotic myocardium caused by calcification was mostly filled by calcified foci, with no cartilage or inflammatory cell infiltration. Analysis of plasma samples from this patient showed that free calcium concentration was higher and PPi concentration was lower than in healthy individuals. Moreover, hepatocellular ABCC6, an important molecule involved in plasma PPi homeostasis, was altered in this patient, being lower than in normal individuals and showing abnormal membrane localization.

Cardiac calcification is generally secondary to myocardial damage (infarction or trauma), with cardiac dystrophic or metastatic calcification resulting from systemic endocrine disruption. The etiological differences between these two subtypes of cardiac calcification are distinct (2). However, both the autopsy and CT-based 3D reconstruction in this patient showed no evidence of dystrophic and metastatic calcification. In addition, increased plasma calcium is one of the pathologic indicators of ectopic calcification, especially metastatic cardiac calcification. The abnormal plasma calcium metabolism is often associated with renal failure, hyperthyroidism or even paraneoplastic hypercalcemia. However, in the present study, the patient had no diagnosis and treatment history of these pathological changes and related diseases. We preliminarily assumed that abnormal levels of vitamin D might be responsible for the elevated plasma calcium in this patient. A literature review identified 46 patients with cardiac calcification (26 dystrophic, eight metastatic, eight idiopathic, and four unknown) over the past 20 years, along with a summary of the etiology and pathological features of these classifications (Table 1). The patient described in the present study was classified as having idiopathic cardiac calcification. Furthermore, calcifications in these patients with myocardial calcification are usually distributed in the ventricular muscles (Table 1, especially the left ventricle), but rarely in the atria. To our knowledge, this patient is the first to present with calcified lesions symmetrically distributed in both atria, a unique physiological location. Additionally, histopathological examination of this lesion showed a large thrombus (Figure 2B, Supplementary Figure S2B), which may have been caused by its unique physiological position. Further examination is needed to determine its specific cause and its relationship to the formation of the calcified lesions.

PPi is a crucial inhibitor of ectopic calcification by regulating the growth of hydroxyapatite crystals (8). Hepatic ABCC6-mediated ATP release is the main source of plasma PPi, suggesting an unanticipated role of the liver in circulating PPi homeostasis (7, 63). A single nucleotide polymorphism in *Abcc6* in several strains of mice, including DBA/2, BALB/c, c3h/He and 129S1/SvJ, results in a natural deficiency of its encoded protein and the susceptibility of these mice to ectopic calcification. The disruption of *abcc6* was also shown to cause ocular calcification and cardiac fibrosis in zebrafish (64). Taken together, these findings indicate that *ABCC6* alteration can result in ectopic calcification. The present study found that the level of expression and the localization of ABCC6 were abnormal in the hepatocytes of this patient. In addition, we further identified 7 heterozygous variants in the patient's *ABCC6* gene. Subsequently, two main questions were raised: pathogenicity analysis of these 7 variants and identification of the genotypes for this patient. For the pathogenicity analysis, long with the further *in silico* analysis, we noticed that the nonsense (c.1132C>T [p.Gln378^*^]) and insert variants (c.196-197insT), which were predicted as the “Pathogenic” variants, can lead to premature termination of ABCC6 translation. These two variants may contribute to the lower ABCC6 content of the patient's hepatocytes. Moreover, along with *in silico* analysis, other 5 missense variants were predicted to be “likely benign,” but these variants have a very low distribution frequency in main population genome databases (<1‰). According to ACMG guidelines, we tended to classify these variants as “uncertain significance (VUS),” which the pathogenicity need further experimental verification. Among these 5 missense variants, the variant of c.373G>A (p.Glu125Lys) has been reported to cause ABCC6 dysfunction (65). Based on *in silico* analysis and literature review, we cannot confirm the pathogenicity of the other four missense variants. However, excepted for c.2645G>A (p.Arg882Lys), other missense variants have been reported and are associated with pseudoxanthoma elasticum(PXE) (66), which indicated that these variants might be pathogenic. The variant of c.2645G>A (p.Arg882Lys) was newly discovered in this patient. Furthermore, interestingly, since the L0 domain is crucial for the membrane localization of ABCC6 (62), the variant of c.841A>G [p.Lys281Glu] might affect the localization of ABCC6 by affecting L0 domain folding but further experimental verification is needed. For the identification of the genotypes for this patient, the co-existence of multiple pathogenic variants could be considered as the compound heterozygous. According to the lower expression instead of completely missing of the immunofluorescence staining for ABCC6, we speculated that the two variants leading to premature termination of ABCC6 might be located on the same allele. And it is unclear whether other variants are distributed on the other allele. However, owing to the *ABCC6* transcript sequence and genotypes of family members of this patient was not available, we also couldn't determine the specific genotypes and inheritance pattern of *ABCC6* variants carried by the patient. Therefore, future studies are required to assess the relationships of these *ABCC6* variants and the inheritance patterns of this gene with cardiac calcifications.

ABCC6 variants/dysfunction is a major pathogenic factor in the development of PXE. Among these identified variants in this patient, several variants have been reported and are associated with PXE. However, it is surprising that this 40 years old patient had no any obvious symptoms of PXE, which is characterized by ectopic calcifications in elastin-rich tissues such as the skin, the Burch's membrane of the retina and the arterial wall (67, 68). Being different from PXE, the calcifications of this patient were concentrated on both sides of the atria, presenting a symmetrical distribution of atrial calcifications. Further researches are needed to determine whether the clinical appearance of this patient is a special phenotype of PXE or whether there is a potential causality between specific ABCC6 variants and cardiac calcifications.

In addition to ABCC6, many other enzymes are involved in the homeostasis of plasma PPi. For example, ATP released from hepatocytes by ABCC6 is hydrolyzed to PPi by hepatic ectonucleotide pyrophosphatase-phosphodiesterase 1 (ENPP1). At the periphery, PPi is further hydrolyzed to Pi by tissue-nonspecific alkaline phosphatase (TNAP). In addition, local ATP levels also depend on the transmembrane protein progressive ankylosis protein homolog (ANKH), a ATP channel/efflux transporter (69). Variants in the *ENPP1, ABCC6* and 5'-nucleotidase ecto (*NT5E*) genes, which are involved in metabolism of PPi and Pi, have been found to predispose to coronary arterial, valvular calcification and other cardiovascular diseases (8, 70–73). Furthermore, overexpression of *Tnap* in endothelium leads to arterial calcification in mice (74). This study focused on ABCC6 in the liver of this patient. By contrast, the other proteins involved in PPi homeostasis have not been investigated thoroughly.

In summary, this study describes a novel patient with idiopathic atrial calcification, with the calcified lesions symmetrically distributed in the patient's atria. The histopathological characteristics of these lesions were assessed by CT-based 3D reconstruction and multiple staining methods. This study also found that the idiopathic atrial calcification in this patient may have been caused by ABCC6 defects in hepatocytes and abnormal plasma PPi levels. Although there is no clear evidence that idiopathic atrial calcification played a role in this patient's death, these findings provided novel clues to the pathogenesis, clinical diagnosis and treatment of idiopathic atrial calcification in future.

## Data Availability Statement

The raw data supporting the conclusions of this article will be made available by the authors, without undue reservation.

## Ethics Statement

The studies involving human participants were reviewed and approved by the fourth military medical university. The patients/participants provided their written informed consent to participate in this study. Written informed consents have been obtained from the next of kin for the publication of any potentially identifiable images or data included in this article.

## Author Contributions

BL and XC: conceptualization, data curation, investigation, statistic analysis, visualization, and writing- original draft. QL, YW, WD, and JZ: autopsy. TC: methodology, data collection, data validation, formal analysis, and resources. YW, KC, and GC: conceptualization, project administration, and writing- editing. All authors contributed to the article and approved the submitted version.

## Funding

This study was supported by the key research and development plan in Shaanxi, Grant/Award Number: 2019SF-059 and 2020SF-204; the Key Innovative Project in Shaanxi, Grant/Award Number: 2021ZDLSF02-02; National Natural Science Foundation of China, Grant/Award Number: 81671476 and 31570906.

## Conflict of Interest

The authors declare that the research was conducted in the absence of any commercial or financial relationships that could be construed as a potential conflict of interest.

## Publisher's Note

All claims expressed in this article are solely those of the authors and do not necessarily represent those of their affiliated organizations, or those of the publisher, the editors and the reviewers. Any product that may be evaluated in this article, or claim that may be made by its manufacturer, is not guaranteed or endorsed by the publisher.
