# Supplementary figures and images for: A Novel Idiopathic Atrial Calcification: Pathologic Manifestations and Potential Mechanism
Source: Front Cardiovasc Med. 2022 Mar 21;9:788958. doi: 10.3389/fcvm.2022.788958 (PMC8978529; doi:10.3389/fcvm.2022.788958)

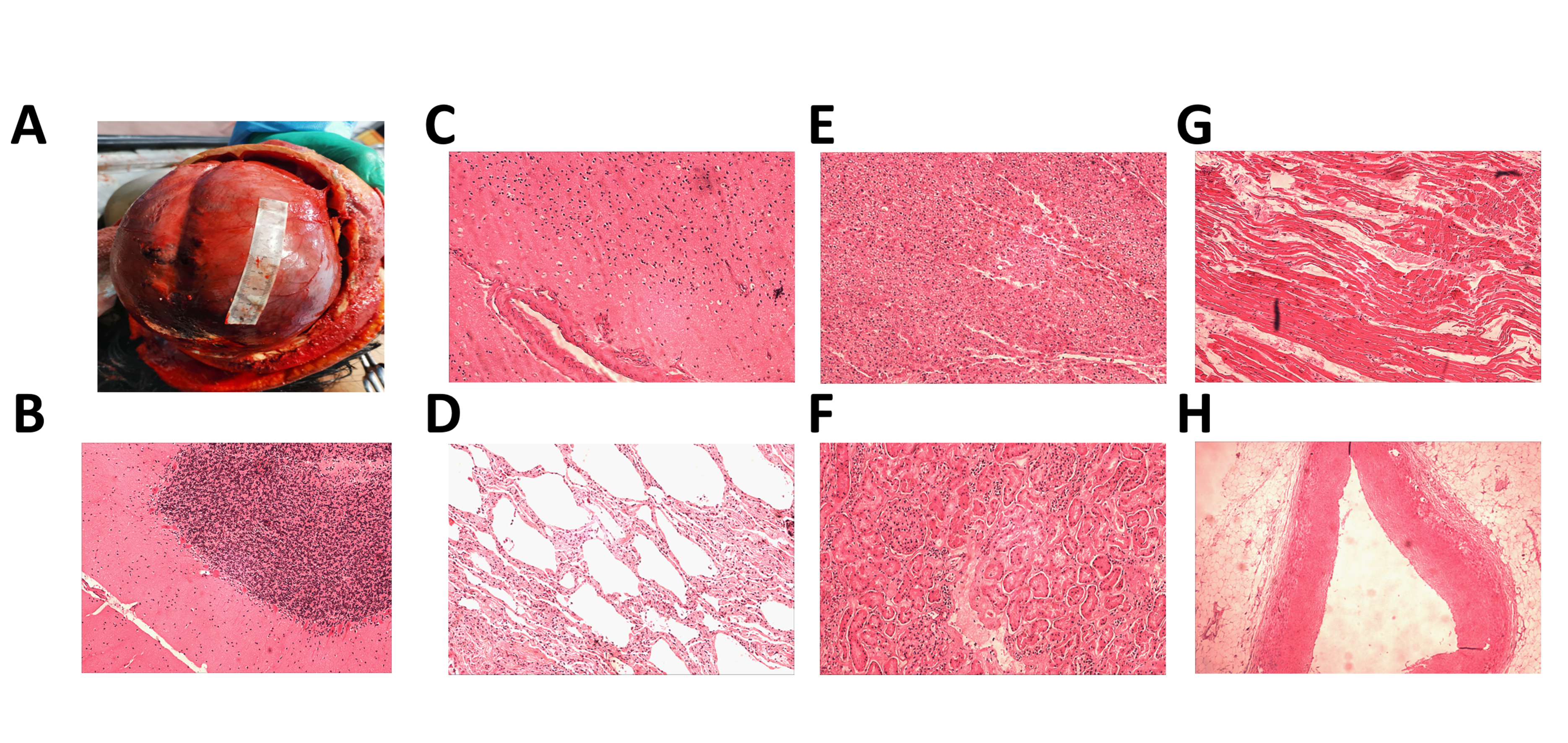

Supplement: Supplementary file 1 [file Image_1.TIF]

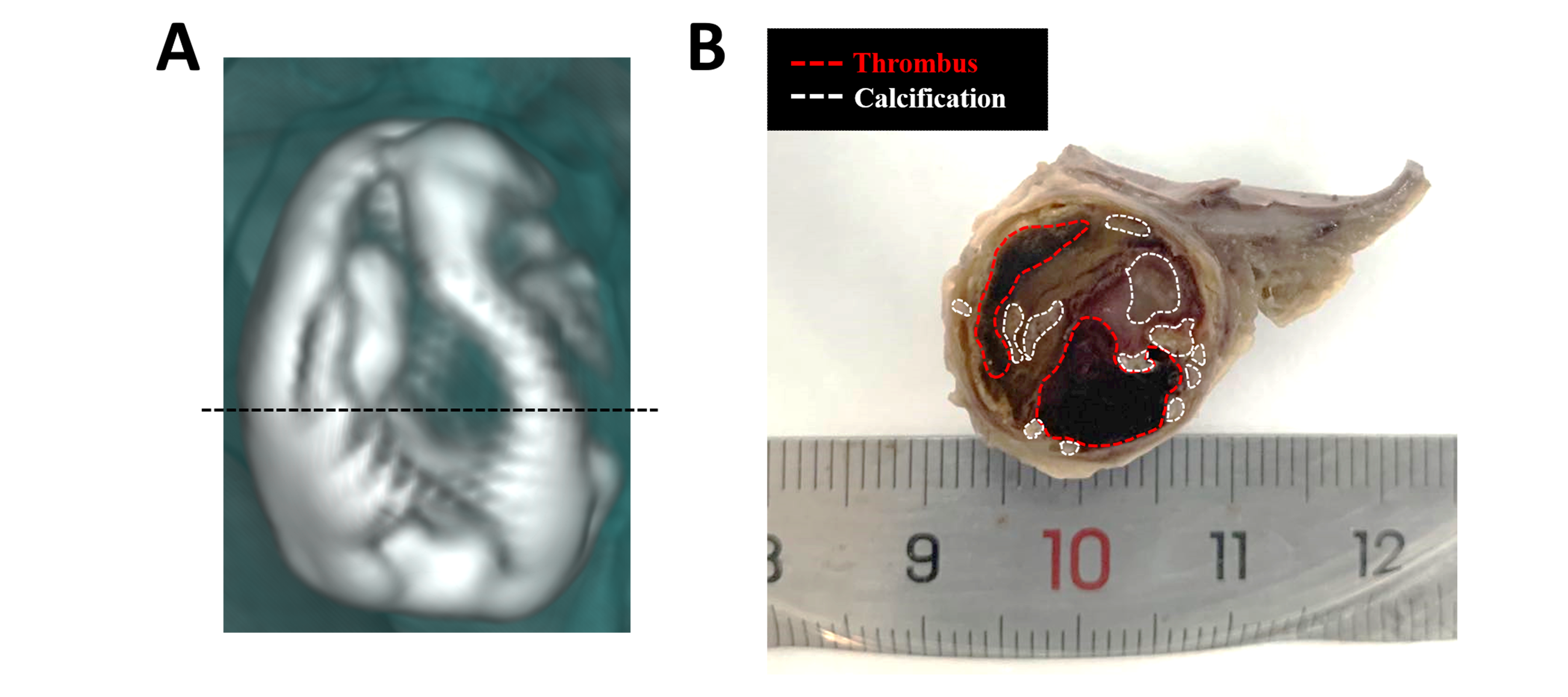

Supplement: Supplementary file 2 [file Image_2.TIF]

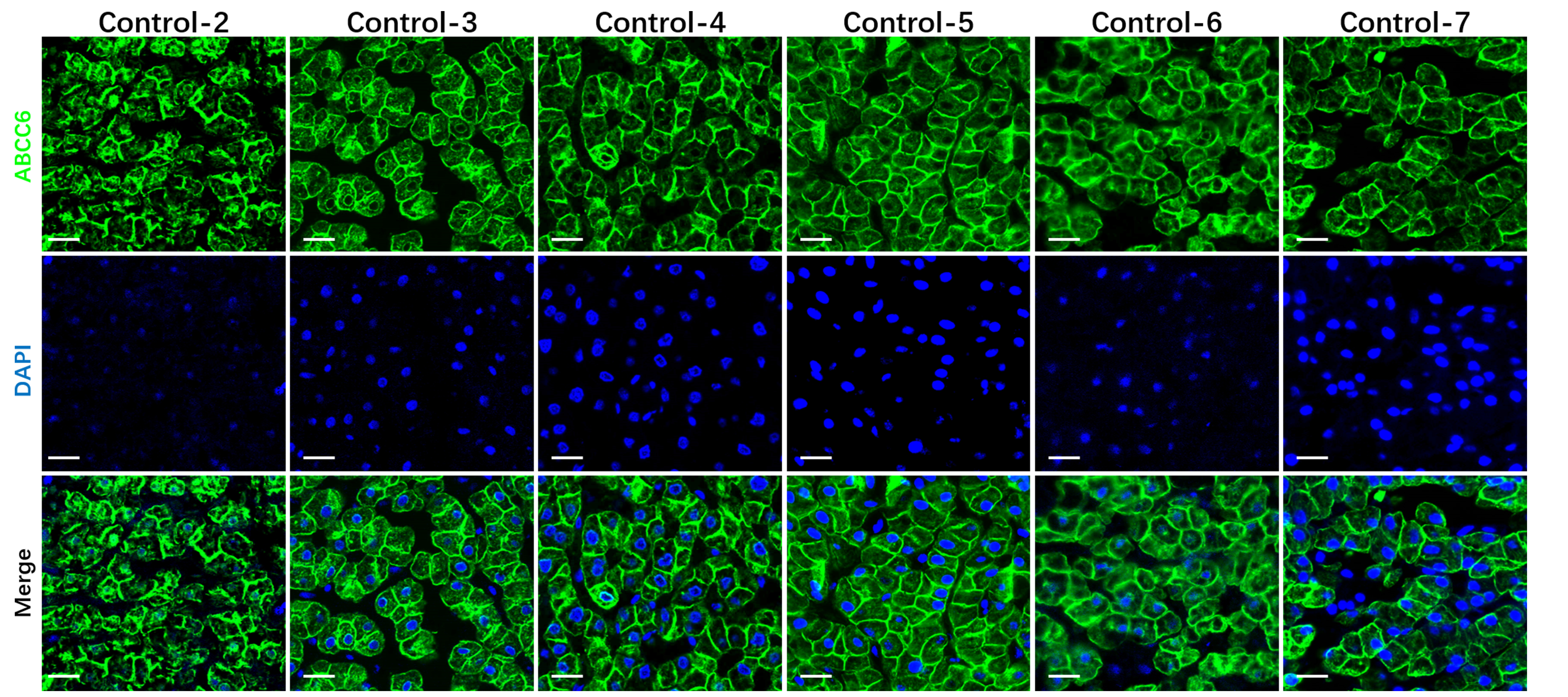

Supplement: Supplementary file 3 [file Image_3.TIF]
