# Supplementary material for: A Novel Idiopathic Atrial Calcification: Pathologic Manifestations and Potential Mechanism
Source: Front Cardiovasc Med. 2022 Mar 21;9:788958. doi: 10.3389/fcvm.2022.788958 (PMC8978529; doi:10.3389/fcvm.2022.788958)
Supplement: Supplementary file 4 [file Data_Sheet_1.docx]

Supplementary Material

**Figure S1 Autopsy examination and histopathology of visceral organs in this patient.**

A. Extensive hemorrhage was observed in the subarachnoid space.

B-H. Histopathologic examination of the (B) cerebellum, (C) cerebrum, (D) lung, (E), liver, (F) kidney, (G) heart and (H) coronary artery. H&E staining, 10×40 magnification in B-G and 10×10 magnification in H.

**Fig. S2 Characterization of a horizontal section of the calcification located in the left atrium.**

A. The black dotted-line indicated the position of the selected horizontal sections of the calcification located in the left atrium.

B. The red dotted-line indicated the region of the thrombus in the calcified lesion. The white dotted-line indicated the regions of calcifications.

**Fig. S3 The distribution of ABCC6 in control group.**

The immunofluorescence of the liver in control-2 to 7. Green indicated ABCC6. Blue indicated cell nucleus. Scale bar= 20 μm.

**Table S1.** List of oligonucleotides used for Sanger sequencing of *ABCC6*

| **Gene** | **Exon** | **Forward Primer (5’-3’)** | **Reverse Primer (5’-3’)** |
| --- | --- | --- | --- |
| *ABCC6* | 1 | CCTCGCCTGTTTTCACCTCC | AAAGAAATCCAACCCGCTGC |
|  | 2 | ACCTAGGGTTAATGTCAGGATGAA | AGATTCCCTTCTACACCCCG |
|  | 3-4 | CATTGGCACGTTGCTAGCTG | GTGCGGGAGTGGATTTTGTG |
|  | 5 | AGAACCACTAGGAAAGCCAGG | TGGCATCGAGTAGAAATGTGGTA |
|  | 6 | AATCGCTTGAACCCAGGAGG | GAAAGCACTGAGGCTGGGAT |
|  | 7 | GCAGACATTAGGTGGGGTTAGTG | TTGAACTCCTGACCTTGTGATCC |
|  | 8 | CACCCATCTCCACTGTTGGG | CCAGACGTATAGGCAGAGGC |
|  | 9 | GACTAGTTTGGGCCGACTGA | AATAAGCAAGGACTGAATGCGT |
|  | 10 | TGGATCCTCAAAGAGCCCTG | GCCTCAGACTTGCCCTAACC |
|  | 11 | TGGAGTTGCTCTGGTTCACG | GTTCGGAGAGCCTATGCTGG |
|  | 12 | CATTATCCCAGGGGCACTCC | GTTTTGATGGACGGGGTGGT |
|  | 13 | CTGGGAGTGGGATCTACGGA | TTTGCTGTACTCTCTGCCCG |
|  | 14 | ACCATCAAGGTCACCACTCG | GCCAGTTTCCAAGTGACACG |
|  | 15 | ACAGAACAAGAACCCTGCGG | CAGGCTGGAAACCTACACCA |
|  | 16-17 | TCCCAGAGCAGGAAAGCTCA | AGAGCTCCTCACTGCCAATC |
|  | 18 | TTGTTGGCTTGAGAGCGAGT | GACTCAAGTGGAAGGGGGAG |
|  | 19 | TGGAGTGCTGTGGTCCAATC | AGTAGGACCCTTCGAGCCTT |
|  | 20 | GAGAGGTTGCTGGAGACGTG | AATGGTTTTGGTTGCCCGC |
|  | 21 | CCAGAGCTCAGTGGCTGTC | TGAGTGAGCGAGCCACTTTC |
|  | 22 | TCGGGGAAATGGTGCTTCTG | AGGACGCAGATCTTTGCCTG |
|  | 23 | GGTGGCCAAGCCATAAGATG | GGAACAGCCCCTAGATGTCC |
|  | 24 | TTTCTCAACCCTGGCTGTCC | GACTGCCTGTGGGATCTAGC |
|  | 25 | AACACCGAGTGTACCCCAGA | GCTCTTGTAGAGCTGCGTGT |
|  | 26 | CAGGTCCTCAGACTGTCCCA | ACCAAGATTTGGCCTGGCTC |
|  | 27 | AGTAGCCACCACCATCTGTT | TGGGTCTGAAAGCTAGGGGA |
|  | 28-29 | ACCTTTACACAATGAGGGATGGA | TAATCCTATCGGGGGAGGCA |
|  | 30 | CATGTGTGGGGATGCCTGAA | CCTCCAGCTCTAACCCGAAG |
|  | 31 | TTAGAGCTGGAGGGGTCCTG | GTGATGGGTGTGGTCGGAAA |
